# Modulations of Cardiac Functions and Pathogenesis by Reactive Oxygen Species and Natural Antioxidants

**DOI:** 10.3390/antiox10050760

**Published:** 2021-05-11

**Authors:** Sun-Hee Woo, Joon-Chul Kim, Nipa Eslenur, Tran Nguyet Trinh, Long Nguyen Hoàng Do

**Affiliations:** 1 College of Pharmacy, Chungnam National University, 99 Daehak-ro, Yuseong-gu, Daejeon 34134, Korea; eslenur45pharm@gmail.com (N.E.); tranctu1994@gmail.com (T.N.T.); dnhlong17@gmail.com (L.N.H.D.); 2 NEXEL Co., 8F 55 Magokdong-ro, Gangseo-gu, Seoul 07802, Korea; jckim@nexel.co.kr

**Keywords:** cardiac Ca^2+^ signaling, ROS, natural antioxidants, mitochondria, cardiac pathogenesis cardioprotective

## Abstract

Homeostasis in the level of reactive oxygen species (ROS) in cardiac myocytes plays a critical role in regulating their physiological functions. Disturbance of balance between generation and removal of ROS is a major cause of cardiac myocyte remodeling, dysfunction, and failure. Cardiac myocytes possess several ROS-producing pathways, such as mitochondrial electron transport chain, NADPH oxidases, and nitric oxide synthases, and have endogenous antioxidation mechanisms. Cardiac Ca^2+^-signaling toolkit proteins, as well as mitochondrial functions, are largely modulated by ROS under physiological and pathological conditions, thereby producing alterations in contraction, membrane conductivity, cell metabolism and cell growth and death. Mechanical stresses under hypertension, post-myocardial infarction, heart failure, and valve diseases are the main causes for stress-induced cardiac remodeling and functional failure, which are associated with ROS-induced pathogenesis. Experimental evidence demonstrates that many cardioprotective natural antioxidants, enriched in foods or herbs, exert beneficial effects on cardiac functions (Ca^2+^ signal, contractility and rhythm), myocytes remodeling, inflammation and death in pathological hearts. The review may provide knowledge and insight into the modulation of cardiac pathogenesis by ROS and natural antioxidants.

## 1. Introduction

Antioxidants are substances that can prevent or slow damage to cells caused by free radicals, unstable molecules that the body produces as a reaction to environmental changes and other stresses. Representative free radicals, endogenously produced by our body, are reactive oxygen species (ROS) and reactive nitrogen species (RNS). A balance between free radicals and antioxidants is necessary for normal physiological function. An unbalanced increase of free radical level adversely alters lipid bilayers, proteins, and DNA and causes many human diseases, including cardiac diseases. Recent evidence additionally shows that ROS and RNS act as ubiquitous intracellular messengers and conduct redox signaling [1,2,3,4]. The present review provides an overview of ROS regulations in cardiac myocytes and their effects on Ca^2+^-signaling proteins and ion transporters/channels that are critical for cardiac rhythm and contraction. In addition, we describe alterations of ROS regulatory mechanisms and their impact on cardiac myocytes under cardiac diseases and natural antioxidants in foods and herbs, which modulate cardiac muscle functions, its pathological remodeling and apoptosis. This review may provide an insight on their potential use to prevent and treat heart failure (HF), ischemia-reperfusion (I–R)-mediated cardiac cell apoptosis, hypertrophic growth and arrhythmias.

## 2. Oxidative Stress and Endogenous Antioxidants in Cardiac Muscle

### 2.1. Cardiac Oxidative Stress and Its Role in Ischemic Injury

ROS are a group of chemical species that comprise at least one oxygen atom in each molecule and display stronger reactivity than molecular oxygen. ROS are classified into free radicals with an unpaired electron (e.g., O_2_^•−^ and OH^•^) and non-radical derivatives (e.g., H_2_O_2_) [5]. The representative RNS is ^•^NO that plays a central role in cardiovascular signaling. Homeostasis of ROS level is required to establish the redox balance of the cell. Excess ROS or fewer antioxidants cause oxidative stress and harmful effects. Potential sources of ROS include the mitochondrial electron transport chain, xanthine oxidase, cytochrome P450-based enzymes and NADPH oxidase (NOX) [5,6]. The bulk of ATP in cardiac cells is generated by oxidative metabolism, which is the sequential passage of electrons from high to low redox potentials down the electron transport chain composed of complexes I to IV [6]. This process results in the active pumping of H^+^ out of the mitochondrial matrix into the intermembranous space. Electrochemical gradient across the inner mitochondrial membrane is then established by the translocation of a proton from the intermembrane space through the Fo/F1 ATPase back into the mitochondrial matrix. This proton translocation is coupled to the phosphorylation of ADP to generate ATP [6]. During this process, O_2_^•−^ are produced via complexes I and III.

The progression of HF, as well as its complications, involves significant mitochondrial remodeling, including Ca^2+^ regulation, ROS or RNS generation, and energy production [6,7,8,9]. In HF, subpopulations of mitochondria around the cell periphery are primarily affected, while interfibrillar mitochondria are less affected [10]. Under pathological conditions, ROS can trigger a burst of ROS production by mitochondria that can lead to apoptotic cell death and inflammatory response. For example, during reperfusion of the ischemic heart, a burst of ROS formation occurs [11,12,13]. Extracellular stimuli, such as stretch, shear stress and angiotensin II (ANG II), often produce O_2_^•−^ via a membrane-associated NOX [14,15,16,17]. NOX family has seven members, NOX1, NOX2, NOX3, NOX4, NOx5, dual oxidase 1 and dual oxidase 2. However, only the NOX1, NOX2, and NOX4 isoforms are expressed in the heart, NOX2 being the predominant isoform in the adult cardiac myocytes [18]. NOX2 is localized in the *t*-tubules of cardiac muscles, which makes it an important regulator of Ca^2+^ signaling at the dyads [19]. It is well-recognized that a higher level of ROS causes oxidative stress in ANG II-associated cardiovascular diseases through activation of NOXs [16,17], mitochondrial dysfunction [20,21,22,23], inflammation [23,24,25,26,27] and the decrease of endogenous antioxidant enzymes [28,29]. Nitrogen oxide synthase (NOS) also contributes to ANG II-related pathological conditions, such as hypertension, atherosclerosis, and diabetes [30] and to stretch signaling in cardiac myocytes [31].

ROS plays a significant role in the pathogenesis of myocardial infarction (MI) and post-MI remodeling in mice [32]. The ROS-mediated nuclear factor kappa-light-chain-enhancer of activated B cells (NF-κB) activation can trigger inflammation and damage through upregulating tumor necrosis factor-α (TNF-α), Bcl-2-associated X protein (Bax) and transforming growth factor β1 (TGF-β1) [33]. NOX2 protein levels as well as NF-κB activity were elevated in cardiac myocytes after acute MI in the infracted area [33,34], supporting that NF-κB is involved in the downstream pathway of ROS. This mechanism may lead to cardiac remodeling. Under myocardial injury, Toll-like receptor 4 (TLR4) is activated, which mediates the inflammatory response [35]. TLR-4 activation requires complex formation with myeloid differentiation protein 2 (MD2). The complex engages with the myeloid differentiation factor 88 adaptor protein (MyD88), which triggers receptor complex interaction with TNF receptor-associated factor 6 (TRAF6) and transforming growth factor-activated kinase 1 (TAK1) [36]. This signaling results in the downregulation of the inhibitor of NF-κB, which further triggers the NF-κB to induce many inflammation mediators [37]. Evidence also suggests that I-R injury in the heart involves necroptosis, a form of programmed necrosis that can be observed downstream of death receptor and pattern recognition receptor signaling under a certain context and triggers inflammatory responses. Necroptosis is known to be triggered by activation of the receptor-interacting protein kinases (RIPK) [38]. Zhu et al. [39] have shown that the RIPK3 is induced in cardiomyocytes with lipopolysaccharide and H_2_O_2_ treatment and in I-R-injury. The induced-RIPK3 representing endoplasmic reticulum (ER) stress leads to cardiomyocyte necroptosis through the increase of intracellular Ca^2+^ level and xanthine oxidase expression. Under these conditions, xanthine oxidase increases cellular ROS and involves mitochondrial permeability transition pore (mPTP) opening [39].

### 2.2. Antioxidant Defense Systems

Intracellular ROS levels are held in check by an intricate array of antioxidant defense systems. Impairment in these defenses and ROS scavenging can also lead to cardiac dysfunction [31,40,41,42,43,44,45]. There are enzymatic antioxidants and a nonenzymatic protection system. The enzymes include catalase, glutathione peroxidase (GSHPx), superoxide dismutase (SOD), and glutaredoxins (Grxs); nonenzymatic antioxidants include vitamins E and C, β-carotene, ubiquinone, lipoic acid, urate, and reduced glutathione (GSH) [46,47,48]. GSH acts as a scavenger of electrophilic and oxidant species either in a direct way or through enzymatic catalysis. GSH is the cosubstrate of GSHPx and allows the reduction of peroxides and the production of GSSG [49]. The GSHPx enzyme is highly expressed in the cytosolic and mitochondrial compartments and is an important protection mechanism in the heart [49]. There are GSH-dependent oxidoreductases that can catalyze *S*-glutathionylation and deglutathionylation of proteins to defend SH groups from oxidation and restore functionally active thiols [50]. The thioredoxin (Trx) system composed of NADPH, thioredoxin reductase (TrxR), and Trx, can provide electrons to thiol-dependent peroxidases (peroxiredoxins) to remove ROS [51]. Peroxiredoxins, placed in different cellular compartments, act as molecular chaperones and phospholipase A2 [52]. Many of these antioxidation pathways are regulated by the transcription factor nuclear factor (erythroid-derived 2)-like 2 (NFE2L2), known as Nrf2 [53,54]. Therefore, Nrf2 exhibits many merits for tissue protection. Under normal conditions, Kelch-like ECH-associated protein 1 (KEAP1) promotes ubiquitination and eventual degradation of Nrf2 [55], while under conditions where the Nrf2-dependent cell defense mechanism is activated, Nrf2 is released from Keap1 and translocates to the nucleus where it binds to conserved antioxidant response element (ARE) sequence, which is followed by induction of expression of an array of cytoprotective gene products, including NAD(P)H quinone oxidoreductase, heme oxygenase-1 (HO-1), GSTs, and glutamate-cysteine ligase catalytic subunit [53,54].

HO-1, in particular, can be induced by numerous stress conditions [56], and thus HO-1 induction is thought to be valuable as a pharmacological target. Many studies have demonstrated the role of HO-1 as an endogenous defense mechanism against cellular injury. The beneficial effect of HO-1 induction on oxidative stress or inflammation is associated with catalyzing the rate-limiting step in the degradation of heme group with its products, ferrous iron (Fe^2+^), carbon monoxide (CO), and biliverdin, which is converted to bilirubin by the biliverdin reductase [57,58,59,60,61]. Excess heme contributes to free radical formation and increases cell damage due to its oxidative and inflammatory properties [62]. It is shown that biliverdin and bilirubin efficiently scavenge chemically generated peroxyl radicals at micromolar concentrations and decrease the peroxidation of low-density lipoproteins in vitro [63,64,65]. Intracellular CO alleviates oxidative damage by modulating mitochondrial function [66].

Pyruvate (2-oxopropanoate), a natural aliphatic carbohydrate produced in cytosol by glycolysis or lactate oxidation, has the capacity to enhance NADPH production and contributes to the glutathione redox state. In fact, this effect increases sarcoplasmic reticulum (SR) Ca^2+^ release [40]. It also has a beneficial effect to regenerate β-adrenergic signaling of ischemically stunned myocardium [40]. It is also known that pyruvate suppresses inflammation in the post-ischemic myocardium by decreasing Ca^2+^ dysregulation and oxidative stress [40]. It has been suggested that infusion of highly concentrated pyruvate solutions improves cardiac mechanical performance and protects the myocardium from ischemic injury [40].

## 3. Regulation of Cardiac Ca^2+^ Signaling by Mitochondria and ROS in Health and Disease

### 3.1. Interaction between Cytosolic Ca^2+^ Signal and Mitochondria

Contraction of cardiac myocytes is elicited by a transient increase in intracellular Ca^2+^ upon action potential firing. This Ca^2+^ increase is caused by a sequence of events (“excitation-contraction coupling”) that includes L-type Ca^2+^ current (I_Ca_)-gated opening of Ca^2+^ release channels (ryanodine receptors, RyRs) and the release of Ca^2+^ from the SR [67,68,69,70]. Laser scanning confocal microscopy has revealed the presence of focal Ca^2+^ release events from RyR clusters in cardiac myocytes (“Ca^2+^ sparks”). The Ca^2+^ sparks are independent of I_Ca_ and voltage and represent unitary properties [71,72,73], indicating that they represent the elementary Ca^2+^ releases for cytosolic Ca^2+^ increase on depolarizations in cardiac myocytes [71,72,73,74,75]. Cytosolic Ca^2+^ is then removed from the cytosol via the SR Ca^2+^-ATPase pump (SERCA2) and sarcolemmal Na^+^–Ca^2+^ exchanger, and sarcolemmal Ca^2+^ pump [76,77].

A large fraction of cell volume (~35%) in cardiac myocytes is occupied by mitochondria [78]. Mitochondria control cellular energy status by regulating O_2_-consuming ATP production. Mitochondrial ATP production allows cardiac contractile function and active transport via the Na^+^–K^+^ pump and SR Ca^2+^ pump. These active transporters are essential for maintaining ionic gradients for Na^+^, Ca^2+^ and K^+^ through the cell membrane and organelle membrane. Appropriate Ca^2+^ handling is also essential in the mitochondrial oxidative phosphorylation, redox balance [79] and for the production of optimal levels of ROS [6] and RNS [80]. However, mitochondrial Ca^2+^ uptake during an action potential has been thought to be very small (about 1%) in ventricular myocytes [81,82]. Nevertheless, a small level of Ca^2+^ taken up by the mitochondria is thought to effectively regulate the oxidative phosphorylation by activating several key enzymes, such as pyruvate dehydrogenase phosphatase, isocitrate dehydrogenase, and α-ketoglutarate dehydrogenase involved in ATP production in the mitochondrial matrix [83,84]. However, both increased and reduced mitochondrial Ca^2+^ levels have been associated with mitochondrial dysfunction [7].

The kinetics of mitochondrial Ca^2+^ uptake during action potential and how much mitochondria contribute to Ca^2+^ signaling are controversial [85]. However, these organelles likely interact with each other in the microdomains in the cardiac myocytes because mitochondria are closely localized to the SR membrane and myofibrils [86,87]. The outer membrane of mitochondria is known to be localized at 37–270 nm away from the SR RyRs [86]. Mathematical modeling has shown that the concentration of Ca^2+^ ([Ca^2+^]) in the narrow space between the mitochondria and SR could reach 10–300 nM when [Ca^2+^] reach 1–2 µM on depolarization [88,89]. Recent experimental evidence supports the role of mitochondrial Ca^2+^ handling in the microdomains of cardiac myocytes [88,89,90,91]. The outer mitochondrial membrane does not have much selectivity for ionic movements. However, the voltage-dependent anion channel (VDAC) 2 in the outer mitochondrial membrane seems to contribute to the decay of Ca^2+^ sparks in the vicinity of RyRs and restrict Ca^2+^ spark expansion in atrial cells under resting conditions [91]. Interestingly, it has been reported that in atrial myocytes lacking transverse (T)-tubules, that peripheral mitochondria and VDAC are involved in regulating Ca^2+^ transients [92]. The level of Ca^2+^ in the space between mitochondria and SR seems to be enough to drive mitochondrial Ca^2+^ uptake through the inner mitochondrial membrane transporter or channels [85]. A major mechanism for Ca^2+^ transport across the mitochondrial inner membrane is known as mitochondrial Ca^2+^ uniporter [6]. Experimental evidence supports that local Ca^2+^ releases (sparks) may activate the low-affinity Ca^2+^ uptake in adjacent mitochondria [90] and thereby showing local mitochondrial matrix Ca^2+^ signals (“Ca^2+^ marks”) [90]. In addition, the size and duration of Ca^2+^ sparks become bigger when the inner mitochondrial Ca^2+^ uptake is inhibited [90]. Ca^2+^ efflux from cardiac mitochondria occurs via the Na^+^–Ca^2+^ exchange (NCXL) [93]. Mitochondrial Na^+^–Ca^2+^ exchange has been shown to regulate mitochondrial Ca^2+^ levels and to connect mitochondrial Ca^2+^ to intracellular Na^+^. Therefore, increased cytosolic Na^+^ concentration that occurs during hypertrophy and heart failure is known to lead to altered redox and metabolism.

### 3.2. Altered Ca^2+^-Signaling Proteins by ROS and Their Pathological Significance

Because of the high-energy demands of the heart, mutations in genes that encode electron transport chain proteins are associated with developing cardiomyopathy [94,95,96]. In addition, it is not surprising that impairment in the electron transport chain altered ATP production with subsequent dysregulations of intracellular Ca^2+^ and increased ROS generation, as well as redox unbalance [97,98]. Cardiac failure, ischemia, and arrhythmia are frequently associated with energy decrease and mitochondrial dysfunction [10,99]. Under pathological conditions of high cytosolic Ca^2+^, mitochondria are capable of taking up large amounts of Ca^2+^, which leads to the opening of the mPTP, a large conductance channel in the inner mitochondrial membrane [100]. The sustained opening of this transition pore is a trigger for cell death [101]. Then, what could be the cellular and molecular basis for ROS-mediated deteriorations of cardiac Ca^2+^ signaling?

In cardiac myocytes, action potential triggers L-type Ca^2+^ channel opening and initiates Ca^2+^ signaling (see above). The pore-forming subunit α_1C_ of the L-type Ca^2+^ channel contains more than 10 cysteine residues, which can undergo redox modification [102]. Thiol oxidizing agents are known to decrease the I_Ca_ [103,104], although there are controversies in the effects of different oxidizing agents on the current in different species [105]. NO enhances I_Ca_ redox-dependently or indirectly inhibits its cGMP-dependently [105].

Ca^2+^ leak through the RyR2 and Ca^2+^ wave under resting conditions increase in cardiac myocytes from HF and atrial fibrillation patients [106,107]. Using murine models are harboring RyR2 mutation that renders the channel leaky (RyR2-S2808D) and a model with RyR2 channels protected against leak (RyR2-S2808A), Santulli et al. [7] have demonstrated RyR2-mediated SR Ca^2+^ leak is associated with increased mitochondrial Ca^2+^ and ROS production, and that constitutive cardiac SR Ca^2+^ leak via RyR2 results in dysmorphic and malfunctioning mitochondria. In this regard, increasing evidence has demonstrated that cardiac RyRs also act as a cellular redox sensor because they have rich free thiol groups in their structure (364 cysteine residues in homotetramer, 21 of which are free on each subunit) [108,109,110]. Oxidation of the free thiols has been thought to activate RyRs in vitro and in situ, and their reductions suppress RyR activity [111,112,113,114]. Treatment of SOD or reducing agents and inhibition of the Complex III in the electron transport chain decrease not only the cytosolic ROS level but also Ca^2+^ spark occurrence in cardiac myocytes [111]. This indicates that basal ROS production and redox balance are responsible for a significant portion of the spontaneous Ca^2+^ spark activity. Application of H_2_O_2_ exogenously at the concentrations of 50–100 μM markedly enhances Ca^2+^ sparks [115,116]. At the concentrations of 200 μM–1 mM exogenous H_2_O_2_ application increased Ca^2+^ sparks and Ca^2+^ transients transiently for 1–3 min, which were followed by suppressing the local and global Ca^2+^ releases in cardiac myocytes [26,117]. Introduction of superoxide by activating xanthine oxidase also biphasically enhance Ca^2+^ spark activity for several min, such that they only transiently enhanced spark occurrence in cardiac myocytes [117,118].

Inositol 1,4,5-trisphosphate receptor (IP_3_R), another Ca^2+^ release channel on the SR membrane, is thought to modulate Ca^2+^ signaling, although the density of IP_3_Rs is much lower than that of RyR2 in cardiac myocytes. In atrial myocytes, they significantly contribute to Ca^2+^ signaling regulation and arrhythmias [118,119,120]. It has been reported only in other cell types, such as hepatocytes and smooth muscle cells, or in vitro system, that oxidizing agents (e.g., thimerosal or oxidized glutathione) stimulate the IP_3_-mediated Ca^2+^ flux [121,122]. It has been shown that cardiac-specific deletion of IP_3_R2 had no major effect on mitochondrial fitness in HF [7].

Ca^2+^ sequestration by SERCA2a in the SR membrane plays a major role in the relaxation of cardiac myocytes. SERCA pump contains 25 cysteine residues, but only 1 or 2 are essential for enzyme action [123]. In contrast to the RyR, thiol oxidizing agents inhibit pump activity, whereas reducing agents protect SERCA from this inhibition [124]. H_2_O_2_ and hydroxyl radicals inactivate cardiac SERCA by interfering with the ATP-binding site on the SERCA [125]. ROS may also inhibit SERCA activity by peroxidation of membrane phospholipids [124]. NO does not appear to alter SERCA activity by *S*-nitrosylation of cysteine residues [126].

The Na^+^–Ca^2+^ exchanger 1, another major pathway of Ca^2+^ removal and homeostasis in cardiac myocytes, consists of 9 transmembrane domains. Disulfide bonds between cysteine residues of different domains of a Na^+^–Ca^2+^ exchanger is thought to be important for its function [127]. Superoxide produced by xanthine/xanthine oxidase reaction, but not by H_2_O_2_ and HOCl, enhanced Na^+^–Ca^2+^ exchange-mediated Ca^2+^ fluxes [128]. ROS burst during reperfusion of the ischemic heart may enhance Ca^2+^ influx mode of Na^+^–Ca^2+^ exchange by an increase of intracellular Na^+^, resulting in Ca^2+^ overload.

Mitochondrial uncoupling using carbonyl cyanide m-chlorophenyl hydrazone (CCCP) or carbonyl cyanide 4-(trifluoromethoxy)phenylhydrazone (FCCP) that disrupt mitochondrial inner membrane potential and mimics ischemic conditions depolarizes the mitochondrial transmembrane potential, thereby inducing reduced Ca^2+^ uptake through the inner mitochondrial Ca^2+^ uniporter [129,130]. This is caused by the removal of the electrical gradient for Ca^2+^ uptake through the mitochondrial membrane. This mitochondrial upcoupling reduces action potential-induced Ca^2+^ transients [131,132] and SR Ca^2+^-loading, which results in cytosolic Ca^2+^ increase at diastole and the increased propensity of spontaneous Ca^2+^ waves [132]. It has been shown using confocal measurement with Mg-fluo-4 that intracellular ATP level decreases under such mitochondrial uncoupling [132]. In addition, I_Ca_ has been inhibited by FCCP [132]. These responses can explain mitochondrial uncoupling-mediated Ca^2+^ transient decease as well as lower SR Ca^2+^-loading.

## 4. Roles of ROS in Cardiac Mechanical Stress Response and Pathogenesis

Changes in the mechanical environment of the heart, caused by each cardiac cycle, alter cardiac excitation and contraction [133,134]. Such mechanical forces in the heart include preload, afterload and shear stress. Increased preload enhances cardiac contractility by Frank-Starling’s law under physiological conditions [133], but high preload makes cardiac cells be largely stretched. Cardiac chambers become enlarged and dilated in HF, valve diseases, and chronic hypertension. Therefore, the stretch stimulus is implicated in developing such diseases. The responses of cardiac myocytes to stretch, including the stimulation of stretch-activated ion channels, have been well documented [135,136,137] and are thought to be an important cellular basis for cardiac remodeling and arrhythmogenesis under congestive HF [134]. The stretch-dependent changes in the cardiac contraction force have biphasic properties: first, a rapid and larger increase in force, and second, a slow increase in force [138,139]. Stretching of the ventricle and atrium is accompanied by increases in Ca^2+^ transient amplitude [140,141,142]. Stretch-induced augmentation of Ca^2+^ transients may result from enhanced unitary Ca^2+^ releases in ventricular myocytes. Stretch is known to activate NOX2 and endothelial isoform of NO synthase (eNOS) activity in the ventricular cells to produce ROS, thereby increasing Ca^2+^ spark occurrences [14,31]. Stretch-induced eNOS activation is known to occur via phosphatidylinositol-3-OH kinase (PI(3)K)-protein kinase B (Akt) signaling [31]. This signaling is a possible downstream signal of the ANG II and endothelin-1 [143,144]. There is a controversy on the role of NOS in the stretch-induced spark enhancement in ventricular myocytes. Some researchers have reported that the blockades of NOS, stretch-activated ion channel, mitochondrial uncoupling do not suppress axial stretch-induced spark enhancement in ventricular myocytes [14].

High shear stress, associated with volume overload, mitral regurgitation and increased afterload, also significantly increased ROS in rat ventricular myocytes through NOX2 [15]. In this shear stress response, a small level of ROS generated via NOX2, in turn, induces bulk mitochondrial ROS generation, which is distinct from the source of ROS in the stretch response of ventricular myocytes [14]. The shear-induced ROS generation enhances resting Ca^2+^ sparks, depolarization-induced Ca^2+^ releases, and SR Ca^2+^-loading. This response also involves an increase of NOS and Na^+^–Ca^2+^ exchanger activity in the prolonged shear stress stimulus [15]. It is still unclear how this shear response plays a role in the pathogenesis of ventricular muscle under pressure- or volume-overload.

In fact, volume- and pressure-overload, associated with hypertension, valvular heart diseases, and heart failure, are clinically associated with atrial fibrillation [145]. Although cellular mechanisms for the mechanical signaling in cardiac myocytes and their clear relevance to specific diseases remain to be fully understood, ROS appears to be a common effector molecule to induce cardiac myocyte remodeling and altered cardiac function. Increased afterload in the transverse aortic constriction (TAC) animal model elicited ROS increase in cardiac cells. The ROS signaling plays a critical role in the alteration of Ca^2+^ signaling and contractility [146]. Activation of NOX has been suggested as a potential player in pressure-overload-induced HF. Various roles for individual NOX isoforms have been reported. In NOX2^y/−^ mice in which pressure overload was induced by TAC, hypertrophy of the left ventricle wall was prevented. However, ROS levels in the myocardium of NOX2^y/−^ mice were increased, which appears to be due to compensation by other NOX isoforms that depend on p22^phox^ and p47^phox^ [147]. ANG II-induced oxidative stress was abrogated in NOX2^y/−^ mice, ROS levels were unchanged after TAC in NOX2^y/−^. Using cardiac-specific NOX4^−/^^−^ mice, it was demonstrated that ROS production under baseline condition is reduced and that after TAC (4 weeks), these mice showed attenuated left ventricular hypertrophy. However, a contrasting finding has been reported using NOX4^−/^^−^ mice and a cardiac NOX4 is overexpressing transgenic model. TAC and MI increased NOX4 expression, but NOX4^−/^^−^ mice showed larger cardiac dilatation and contractile dysfunction compared with wild-type (WT) mice, and NOX4 transgenic mice developed less hypertrophy and fibrosis compared with WT mice [148].

## 5. Exogenous Natural Antioxidants to Protect Cardiac Muscle from Oxidative Stress

### 5.1. Flavonoids

Flavonoids are a group of polyphenolic compounds diverse in chemical structure and characteristics. Flavonoids are classified into four predominant classes, 4-oxoflavonoids (flavones and flavonols), isoflavones, anthocyanins, and flavan-3-ol derivatives (tannins and catechin) [149,150]. They are well-known as antioxidants, free radical scavengers, and chelators of divalent cation [151,152]. SR Ca^2+^-ATPase pumps are known to be inhibited by various hydrophobic molecules that can be derived from natural products, such as thapsigargin [153], curcumin [154], and the flavonoid quercetin [155]. Some flavonoids can bind to nucleotide-binding sites of the SR Ca^2+^-ATPase pump and change its activity, which can result in apoptosis via increased cytosolic Ca^2+^ level and initiation of Ca^2+^-dependent mitochondrial pathway [156].

*Catechin* [(2R,3S)-3′,4′,5,7-tetrahydroxyflavan-3-ol] has been proven to effectively suppress lipid peroxidation and scavenge free radicals [157,158]. Several previous reports have shown that green tea (*Camellia sinensis*) containing catechin (specifically epigallocatechin-3-gallate (EGCG)) has a cardioprotective effect [159,160]. It has been shown that EGCG protects I-R-induced cardiac myocytes apoptosis by decreasing phosphorylation of STAT-1, which is a transcription factor involved in the promotion of apoptosis [161,162]. Sheng et al. [163] have also shown that EGCG inhibits cardiac myocytes apoptosis and oxidative stress in pressure overload-induced hypertrophic hearts [162]. *Luteolin* (3′, 4′, 5′, 7′-tetrahydroxyflavone), one of the most prevalent flavones, is known to inhibit apoptosis by upregulating Akt in a simulated I-R model [163]. It also increases Bcl-2 expression and the Bcl-2 to Bax ratio and reduces Bax expression. The SERCA2a activity has been shown to be improved by luteolin via the activation of the PI(3)K/Akt signaling pathway with an increase of phosphorylated Akt. Luteolin does not seem to change the expression of SERCA2a at the protein level [164]. In adult rat cardiac myocytes, luteolin is known to improve contractile function and reduce apoptosis after I-R injury [165].

*Quercetin* (2-(3,4-dihydroxyphenyl)-3,5,7-trihydroxy-4*H*-chromen-4-one) is one of the most abundant dietary flavonoids, and it contains a polyphenolic chemical substructure that prevents oxidation in the oxidative chain reactions by scavenging free radicals, thereby preventing inflammation, hypertension and ischemic heart diseases [166,167]. It is known to protect cardiac myocytes from myocardial injury under doxorubicin treatment. This substance also reduces ROS generation and increases the endogenous antioxidant enzymes and non-enzymes (see above) [168]. Santos et al. [169] have shown that quercetin increases I_Ca_ under β-adrenoceptor stimulation and intracellular Ca^2+^ transient without changing Ca^2+^ sparks [112]. *Anthocyanins* are a water-soluble pigment subgroup mainly found in flavonoid groups that are ranging from red to blue colors in many kinds of plants, flowers, and seeds, etc. [169]. The strength of antioxidation among anthocyanins is controlled by their differences in chemical structure, such as the number and location of conjugation groups, hydroxyl groups, glycosylation, donor electrons in the ring structure, as well as the aromatic group’s capacity to sustain the disappearance of electrons [170,171]. Shaughnessy et al. [172] have shown that systolic blood pressure can be decreased by a blueberry-enriched diet in spontaneously hypertensive stroke-prone rats [172]. Other previous studies also have shown that consumption of blueberries-containing food can suppress hypertension and prevent cardiovascular disease. The blueberries diet has been suggested to alter aortic contractility via modulation of the NO metabolic pathway. Other reports suggest that its vasodilator effect is dependent on the endothelium [173,174,175,176].

Honey from honeybee (*Apis mellifera*) contains acacetin and kaempferol that are effective in the heart. *Acacetin* (a 4′-*O*-methylated flavone), a radical scavenging flavonoid, inhibits ultrarapid delayed rectifier K^+^ current and prolongs action potential duration in human atrial myocytes [177]. It also blocks the acetylcholine-activated K^+^ current in guinea-pig cardiac myocytes [177]. Based on these mechanisms, acacetin has been suggested as an atrium-specific anti-atrial-fibrillation agent. It is known that *kaempferol*, 3,4′,5,7-tetrahydroxyflavone, inhibits endothelial dysfunction and activation, resulting in reductions of cardiac fibrosis and left ventricular diastolic dysfunction in pathological condition of ANG II infusion [178]. In addition, this can protect cardiac cell apoptosis caused by I-R injury [179]. *Rutin*, also called rutoside and sophorin, is the glycoside combining the flavonol quercetin and the disaccharide rutinose and is a citrus flavonoid found in tea (*Camellia sinensis*), buckwheat (*Fagopyrum esculentum*), tobacco (*Nicotiana tabacum*) and stink beans (*Parkia speciosa*). Rutin has cardioprotective effects under I-R injury and also decreases cardiac hypertrophy induced by ANG II via attenuating intracellular Ca^2+^ increase. Rutin is known to upregulate sirtuin 1 in vitro and in vivo [180,181,182]. It has been suggested that rutin decreases cardiomyocyte hypertrophy induced by ANG II via the suppression of an increase of intracellular Ca^2+^ level [183].

*Isorhamnetin*, an O-methylated flavonol found in the Chinese herb *Hippophae rhamnoides* L., has suppressed cardiac hypertrophy by blocking the PI3K-Akt pathway [184]. In H9c2 cardiac cells, isorhamnetin has been shown to protect I-R injury by decreasing apoptosis and oxidative stress [185]. In the same cardiac cell line, this compound also has reduced ROS level, inactivated extracellular signal-regulated kinase (ERK) and inhibited H_2_O_2_-induced intrinsic apoptotic pathway [186]. *Rhamnetin* (O-methylated flavonol) from spiraea has cardioprotective effects in miconazole-stimulated H9c2 cardiac cells through ROS reduction [187] and in ischemia-induced cardiac injury [188]. Similar cardioprotective effects have been observed with *apigenin* (4′,5,7-trihydroxyflavone) from the flowers of chamomile. It has beneficial effects to prevent cardiac cell death under I-R injury through the PI(3)K-Akt pathway [189]. It suppresses cardiac hypertrophy and downregulates hypoxia-inducible factors in rats [190].

*Baicalein* (5,6,7-trihydroxyflavone) from the root of *Scutellaria baicalensis* Georgi [191] alleviates E3 ubiquitin–protein kinase (MARCH5) expression to inhibit apoptosis caused by oxidative stress in cardiac myocytes [192,193]. In addition, it has been reported that it protects cardiac hypertrophy in mice through initiating autophagy and repressing oxidative stress [194]. It is shown that baicalein suppresses the lipopolysaccharide-induced NO production in RAW 264.7 mouse macrophages in vitro [195] and reduced plasma NO levels leading to improved vasoreactivity, blood pressure, and survival rate in septic rats [196]. Lee and colleagues investigated the protective effect of baicalein related to HO-1 against myocardial dysfunction caused by lipopolysaccharide-induced endotoxemia in rats [197]. According to this study, baicalein seems to improve myocardial contractility in lipopolysaccharide-induced sepsis, which may be related to reductions in oxidative stress by induction of HO-1 protein and suppression of superoxide anion formation.

*Silymarin*, a standardized extract of the milk thistle seeds containing a mixture of flavonolignans, can decrease abnormal growth of cardiac myocytes via downregulation of epidermal growth factor [198]. *Naringin*, a flavanone-7-*O*-glycoside, contained in *Drynaria fortune, Citrus aurantium* L. and *Citrus medica* L., is known to protect cardiac myocytes against hyperglycemia-induced injuries in vitro and in vivo [199]. It ameliorates hypoxia/reoxygenation-induced ER stress-mediated apoptosis in H9c2 cells via activating transcription factor 6 (ATF6), inositol-requiring enzyme1α (IRE1α) and ERK signaling activation [200]. A previous report has shown that naringin improves mitochondrial function and reduces cardiac damage following I-R injury via the AMP-activated protein kinase (AMPK)-sirtuin 3 signaling pathway [201].

**Figure 1:**
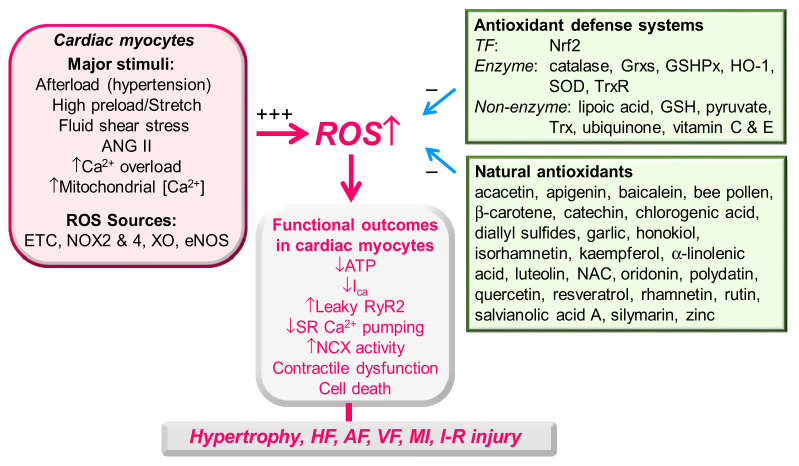
Effective substances and mechanisms to decrease or increase ROS level and functional outcomes of redox unbalance in cardiac myocytes and heart. Redox unbalances by overproduction of ROS via fewer antioxidants or excess ROS-producing stimuli may result in dysregulation of Ca^2+^ signaling and metabolism (“functional outcomes”) and are associated with the pathogenesis of cardiac diseases. ETC., electron transport chain; NCX, Na^+^–Ca^2+^ exchanger; XO, xanthine oxidase.

Figure 1 shows a summary on effective substances and mechanisms to decrease or increase ROS level and functional outcomes of redox unbalance in cardiac myocytes and heart.

### 5.2. Non-Flavonoids

*N-acetylcysteine (NAC)* is a popular antioxidant that possesses a sulfhydryl group, which acts as a source of cysteine to glutathione synthesis and is used as a generic medication to treat acetaminophen overdose. It is contained in onion (*Allium cepa*). This compound is commonly used in the research laboratory to decrease ROS levels and test its role in biological responses. In the rat ventricular myocytes, it has been shown that NAC slightly suppresses resting Ca^2+^ spark occurrence [15]. In pressure-overloaded rats, NAC has suppressed myocardial fibrosis during the transition from compensated left ventricular hypertrophy to HF [202]. Myocardial total glutathione level appears to be upregulated by NAC treatment, while mitogen-activated protein kinase (MAPK) signaling is downregulated by NAC [202].

*Zinc* is an essential mineral largely contained in meats and oysters that is required for various cellular functions, and it has a critical antioxidant action. Metallothionein (Zn^2+^-binding protein) expression increases at the sites of cardiac injury, which allows local accumulation of Zn^2+^, thereby accelerating gene transcription and wound healing. Zn^2+^ insufficiency can delay this process under ischemic conditions [203,204,205,206,207,208,209]. It is known that Zn^2+^ transport into the cells is achieved by Zn^2+^ transporters and L-type Ca^2+^ channels. In addition, the Zn^2+^ transporters can be activated by oxidative stress. The level of cytosolic free Zn^2+^ increases by NO, derived from endothelial NOS [210].

*Resveratrol* (3,5,4′-trihydroxy-*trans*-stilbene) is a phytoalexin produced by several plants in response to injury or when the plant is under attack by pathogens. It enhances the SOD activity and eNOS activity and increases the level of glutathione [211]. Pretreatment with resveratrol from red wine (*Vitis vinifera*) and blueberries (*Cyanococcus*) is reported to reduce infarct size and both tachycardia and bradycardia after myocardial infarction [212]. Resveratrol improves cardiac function via increases of sirtuin 1 (SIRT1) activity and AMPK activity [213]. The long-term dietary supplement of resveratrol has improved ventricular systolic function as well as an atrioventricular coupling in chronic HF rats derived from permanent coronary artery ligation [214]. The cardiovascular protective effects by resveratrol also have been recently reviewed by Carrizzo et al. [167]. *Polydatin*, a resveratrol glucoside found in Hu-zhang (*Polygonum cuspidatum*) and red wine, is one of the antioxidants showing anti-inflammation and anti-platelet coagulation. This compound can increase cardiac contraction [215,216] and also has a cardioprotective effect against I-R injury and pressure-overload-induced ventricular remodeling [184]. In rat cardiac myocytes, SOD, NOS and NO were upregulated by polydatin [185]. A more recent study has shown that polydatin suppresses I_Ca_ and Ca^2+^ transients and that it increases cardiac RyR activity. This compound increases contraction by enhancing myofilament Ca^2+^ sensitivity via NO production [186].

*Honokiol* (3′,5-di(prop-2-en-1-yl)[1,1′-biphenyl]-2,4′-diol), a lignan isolated from the bark of magnolia trees, has the capacity to activate mitochondrial sirtuin 3 that inhibits ROS production, thereby locking cardiac hypertrophy in mice [217]. Another group also has reported that this compound improves mitochondrial function and protects against doxorubicin-induced cardiotoxicity [218]. Danshen (*Salvia miltiorrhiza*) component *salvianolic acid A*, a stilbenoid ((R)-3(3,4-dihydroxyphenyl)-2-(((E)-3-(2-((E)-3,4-dihydroxystyryl)-3,4-dihydroxyphenyl)acryloyl)oxy)propanoic acid), is reported to have inhibitory effects on ventricular fibrillation and lipid peroxidation [219]. This compound has significantly attenuated cardiac dysfunction and injury induced by isoproterenol and enhanced mitochondrial respiratory function [220]. In addition, it has shown protective effects against inflammatory injury by modulating forming MD2-TLR4-MyD88 complex and TLR4-TRAF6-NF-κB signaling pathway in acute MI rats [221]. This compound also suppressed L-type Ca^2+^ channels, contraction, and Ca^2+^ transients in adult rat cardiac myocytes [222].

*α-linolenic acid* is normally found in seeds, nuts and oils, and has beneficial effects of decreasing the risk of cardiac arrhythmias, especially ventricular fibrillation, and protect against the risk of HF and cardiac hypertrophy [223]. Under ischemia and I-R, α-linolenic acid can protect cardiac cells from the apoptosis process by reducing the production of specific pro-apoptotic oxidized phosphatidylcholine species. Thus, the pro-apoptotic oxidized phosphatidylcholine species are thought to be a potential target to protect the heart from ischemic damage [224]. *β-carotene* (a terpenoid strongly colored red-orange pigment), contained in carrots, spinach and tomatoes, has a good effect of reducing the possibility of acute MI. β-carotene can significantly reduce cell death and apoptosis induced by “advanced glycation end products”, which are abundant in aged persons and in the patients with diabetes mellitus, degenerative diseases, and chronic kidney disease, and are the main cause of diabetic cardiomyopathy. In addition, a recent report demonstrated that it decreases the production of intracellular ROS, antioxidative enzyme, hyperactive ER stress and autophagy via the activation of the PI(3)K/Akt/mammalian target of rapamycin (mTOR) pathway in H9c2 cells [225].

*Chlorogenic acid*, the ester of caffeic acid and (−)-quinic acid found in *Eucommia ulmoides,* is known to be a free radical scavenger and suppress the activation of the mitogen-activated protein kinase kinase (MEK)/ERK signaling pathway and myocardial I-R injury in rats [226]. This substance modulates the protein-B expression in cardiac myocytes following hypoxia-reoxygenation through the ROS signaling pathway [227]. It is also known that chlorogenic acid shows cardioprotective effects via directly suppressing the activation of the NF-κB and c-Jun N-terminal kinase (JNK) pathway in the TAC mouse model. Therefore, this component may be useful for the prevention of HF [228].

*Oridonin* (7,20-epoxy-1α,6β,7α,14R-tetrahydroxy-kaur-16-en-15-one; a diterpenoid), the major active ingredient of the traditional Chinese medicinal herb *Rabdosia rubescens,* has been suggested to have antihypertrophic effects in cardiac muscle [229]. It has been demonstrated that oridonin treatment increases the expression of myocardial HO-1 in pressure-overloaded heart muscles and that it also limits ROS generation in these hearts with an increase of myocardial SOD and GPx [229]. Furthermore, Zhao et al. [230] have demonstrated that *cinnamaldehyde*, one of the main bioactive constituents isolated from *Cortex cinnamomi*, can ameliorate cardiac dysfunction induced by lipopolysaccharides in rats through suppression of TLR4-NOX4 signaling, ROS production, and autophagy.

*Bee pollen*, a substance packed by worker honey bees during collection, is obtained from field-gathered flower pollen and honey, agglutinated into pellets with bee saliva. It comprises proteins, simple sugars, minerals and vitamins, and fatty acids. Due to the numerous bioactive compounds, bee pollen has been reported to have many pharmacological properties, including antioxidative effects [231,232,233,234]. According to Zhang et al.’s study, *Schisandra chinensis* bee pollen extract (SCBPE) possesses the most robust total antioxidant capacity among ten kinds of bee pollens, evaluated by radical scavenging activity, Trolox equivalent antioxidant capacity, and reducing power [235]. The cardioprotective effect of SCBPE against ROS attack also has been demonstrated in animal model experiments. Shen et al. [231] studied the changes in the expression of Nrf2 and HO-1 proteins by SCBPE in cardiac tissues of rat MI model induced by isoproterenol. After administrating SCBPE to rats for 30 days, the protein expression of Nrf2, HO-1, and Bcl2 (apoptosis regulator) in the heart increased in the SCBPE groups, while Bax protein (apoptotic activator) and pathological cardiac phenotype were reduced compared to the control group [231].

*Garlic* (*Allium sativum*) is widely known as a natural product with plenty of beneficial effects, such as antioxidative [236], antibacterial [237], lipid-lowering [238], and antitumor [239] activity. Notably, it is getting more attention for its powerful cardioprotective effects [236]. The organosulfur compounds are the primary active ingredients in garlic. Diallyl sulfide (DAS), diallyl disulfide (DADS), and diallyl trisulfide (DATS) have been studied, and their antioxidative effects are more substantial with a higher number of sulfur atoms (DATS > DADS > DAS) [240,241]. In Yu et al.’s study, DATS treatment markedly improved left ventricular systolic pressure and reduced myocardial infarct size, serum creatine kinase, lactate dehydrogenase activities, and the myocardial apoptosis in type 1 diabetic rats. They showed that these effects were partly due to the activation of the Nrf2/HO-1 antioxidant signaling pathway [242].

Table 1 categorizes natural antioxidants according to their target diseases in the heart, and summarizes their effects and action mechanisms. 

## 6. Conclusions

In cardiac myocytes under physiological conditions, Ca^2+^ signaling on regular action potentials contributes to mitochondrial Ca^2+^ signal, which, in turn, regulates oxidative phosphorylation of mitochondria to produce ATP with generating a small amount of ROS. ATP, produced by the mitochondria, plays a key role in maintaining cytosolic concentrations of Na^+^, Ca^2+^, and K^+^ by driving Na^+^–K^+^ pump, SR Ca^2+^ pump and Na^+^–Ca^2+^ exchange during beating. This is mandatory for generating normal action potentials and subsequent Ca^2+^-induced Ca^2+^ release to trigger contraction and relaxation during each cardiac cycle that determines arterial blood pressure. Ischemia and pressure- or volume-overload induce initial cardiac hypertrophy, which is accompanied by HF and arrhythmias. It is thought that remodeling and death of cardiac myocytes by bulk ROS generation via NOX (NOX2 and NOX4) and mitochondria and by Ca^2+^ overload during the oxygen deprivation and mechanical stresses play key roles in contractile failure and arrhythmias. Natural antioxidants may serve as an alternative way to prevent such oxidative stress-dependent cardiac pathogenesis. Antioxidants that are effective for ischemia and reperfusion injury, associated with inflammation and apoptosis/necroptosis, have been discovered from red wine (resveratrol), fruit, such as blueberries (quercetin), herbs, including green tea (catechin), chamomile (apigenin), and *Citrus* plants (naringin), vegetables, such as carrot (β-carotene), celery (luteolin), and garlic (diallyl sulfides), and buckwheat (rutin), honey (kaempferol), bee pollen extract, and medicinal plants, including *Polygonum cuspidatum*, Hu-zhang (polydatin), *Hippophae rhamnoides* (isorhamnetin), danshen (salvianolic acid A), and *Eucommia ulmoides* (chlorogenic acid). α-linolenic acid in the nuts and seeds has shown anti-arrhythmic and antihypertrophic effects. Resveratrol and salvianolic acid A have anti-arrhythmic effects and cardiotonic effects in HF. Quercetin decreases Ca^2+^ currents and is beneficial for HF. Interesting to note is that acacetin from honey has anti-atrial-fibrillation effects.

In fact, clinical investigations on the effects of natural antioxidants significantly increased in recent years, but they mostly focus on blood and vascular function. A randomized clinical trial (RCT) has indirectly shown that oral supplementation of resveratrol in patients with stable angina may be cardioprotective because it decreases the inflammatory marker, brain natriuretic peptide and lipid marker [243,244]. The RCT recently demonstrated that quercetin has beneficial effects on blood pressure, blood lipids and endothelial function [245]. In addition, chlorogenic acid and its physiological metabolites improved human vascular functions [246]. Supplementation with green tea catechin extract or α-linolenic acid significantly reduced circulating total cholesterol concentrations [247,248]. The RCTs investigating the effects of flavonoid-rich foods on cardiovascular function have shown that most compounds, except pro-anthocyanidins, are extensively metabolized and display poor bioavailability [245,249]. Nevertheless, it was recently suggested that flavonoid metabolites could be more bioactive than their precursors [245]. Further in vivo research and clinical trials need to be initiated to further validate the cardio-effective natural antioxidants in medicinal applications for cardiac hypertrophy, atrial fibrillation, MI and congestive HF.

## Figures and Tables

**Table 1:** Exogenous natural antioxidants to modulate cardiac function and pathogenesis.

Cardiac Disease	Antioxidant	Source	Effects/Mechanisms	References
Arrhythmias	Acacetin	Honey	Anti-AF, ↓I_Kur_	[177]
	α-Linolenic acid	Seed, nut, and their oil	Anti-VF, Anti-HF	[223]
	Resveratrol	Red wine, blueberry	Anti-arrhythmias	[212]
	Salvianolic acid A	Danshen	Anti-VF	[221]
HF/Contractile dysfunction	Polydatin	Hu zhang (*polygonum cuspidatum*)	↓I_Ca_, ↑RyR activity, ↑Myofilament Ca^2+^ sensitivity	[186,215,216]
	Quercetin	Oak, blueberry	↓I_Ca_, ↑Ca^2+^ transient	[112]
	Luteolin	Celery, parsley	↑Contraction, ↑SERCA	[165]
	NAC	Onion	↓HF	[202]
	Resveratrol	Red wine, blueberry	↑Contraction	[214]
	Salvianolic acid A	Danshen	↓I_Ca_, Ca^2+^ transient, contraction	[222]
Hypertrophy	Rutin	Tea, buckwheat, tobacco	↓Intracellular Ca^2+^	[183]
	NAC	Onion	↓MAPK	[202]
	α-Linolenic acid	Seeds, nuts, and their oils	?	[223]
	Silymarin	Milk thistle	↓EGFR	[194]
	Honokiol	Magnolia tree bark	↑Mitochondrial sirtuin 3	[217]
	Isorhamnetin	*Hippophae rhamnoides* L.	↓PI3K-Akt	[184]
	Apigenin	Chamomile	?	[190]
	Oridonin	*Rabdosia rubescens*	?	[229]
MI I-R injury	Resveratrol Polydatin	Red wine, blueberry Hu-zhang	↑AMPK-sirtuin 1, ↓apoptosis	[184,213]
	Luteolin	Celery, parsley	↑Akt, ↓apoptosis	[163]
	β-carotene	Carrots, spinach, tomatoes	↑p-Akt, ↓apoptosis	[225]
	EGCG/catechin	Green tea	↓p-STAT-1, ↓apoptosis	[161,162]
	Kaempferol	Honey	↓Inflammation	[179]
	Quercetin	Oak, blueberry	↓Inflammation	[166,167]
	Isorhamnetin Rhamnetin	*Hippophae rhamnoides* L. *Spiraea*	↓ROS, ↓ERK ↓ROS	[185,187,188]
	Apigenin	Chamomile	↑PI3K-Akt	[189]
	Chlorogenic acid	*Eucommia ulmoides*	↓MEK/ERK	[209]
			Protein B	[210]
	Rutin	Tea, buckwheat, tobacco	↓p-Akt	[180,181,182]
			↑Sirtuin 1	[181,182]
	DATS, DADS, DAS	Garlic	↑Nrf2/HO-1	[242]
	Baicalin	*Scutellaria baicalensis*	↑Autophagy, ↑MARCH5 ↑HO-1	[192,193,194,197]
	Naringin	Citrus	↑ATF6-IRE1α-ERK ↑AMPK-sirtuin 3	[196,197]
	Zinc	Meat, oysters	↓Oxidation	[203,204,205,206,207,208,209]
	Salvianolic acid A	Danshen	↓MD2-TLR4-MyD88, ↓TRAF6-NF-κB	[221]
	SCBPE	Bee pollen	↑Nrf2, HO-1, and Bcl2	[231]

AF, atrial fibrillation; Akt, protein kinase B; EGFR, epidermal growth factor receptor; ERK, extracellular signal-regulated kinase; HF, heart failure; I_Ca_, L-type Ca^2+^ current; I_Kur_, ultrarapid delayed rectifier K^+^ current; I-R, ischemia-reperfusion; MEK, mitogen-activated protein kinase kinase; MI, myocardial infarction; RyR, ryanodine receptor; SCBPE, *Schisandra chinensis* bee pollen extract; VF, ventricular fibrillation; DAS, Diallyl sulfide; DADS, diallyl disulfide, DATS, diallyl trisulfide. “?” indicates unknown.
